# Solving the multicommodity flow problem using an evolutionary routing algorithm in a computer network environment

**DOI:** 10.1371/journal.pone.0278317

**Published:** 2023-04-19

**Authors:** Noel Farrugia, Johann A. Briffa, Victor Buttigieg

**Affiliations:** Department of Communications and Computer Engineering, University of Malta, Msida, Malta; Jinan University, China, HONG KONG

## Abstract

The continued increase in Internet traffic requires that routing algorithms make the best use of all available network resources. Most of the current deployed networks are not doing so due to their use of single path routing algorithms. In this work we propose the use of a multipath capable routing algorithm using Evolutionary Algorithms (EAs) that take into account all the traffic going over the network and the link capacities by leveraging the information available at the Software Defined Network (SDN) controller. The designed routing algorithm uses Per-Packet multipath routing to make the best use of the network’s resources. Per-Packet multipath is known to have adverse affects when used with TCP, so we propose modifications to the Multipath TCP (MPTCP) protocol to overcome this. Network simulations are performed on a real world network model with 41 nodes and 60 bidirectional links. Results for the EA routing solution with the modified MPTCP protocol show a 29% increase in the total network Goodput, and a more than 50% average reduction in a flow’s end-to-end delay, when compared to OSPF and standard TCP under the same network topology and flow request conditions.

## Introduction

One of the major drawbacks of computer networks using a distributed architecture is their low efficiency caused by the lack of routing solutions aware of the entire network status. In a distributed architecture network, every routing device contains its own independent control plane; each routing device takes independent routing decisions based on information local to the device. A distributed network can improve the routing decisions taken by individual components by constantly sharing a snapshot of the current global network status. However, due to the impracticality of such a solution, distributed network architectures resort to either single path routing algorithms or very simple multipath solutions such as Equal Cost Multipath Routing (ECMP) [1]. In ECMP, flows are distributed over paths with equal cost where a hash of the packet header, not the current network state determines the path taken [2]. Improving the network resource usage efficiency requires the use of routing algorithms with access to an accurate and up-to-date snapshot of the global network status. A centralized network architecture, such as that provided by a Software Defined Network (SDN) [3], provides such information by moving the control plane to a central location. While SDN and control plane centralization is often considered a recent invention, the centralization of the network control plane is anything but; [4] gives an interesting insight on the origins of SDN. As an example, the improved efficiency enabled by SDN has been vital in the deployment of Google’s B4 international network that would have otherwise been unfeasible [5].

In this work, we aim to increase the network’s efficiency by focusing on route optimization for an already deployed network. An alternative would be to design a network topology from scratch that lends itself to the use of multipath and reduced power consumption to operate such a network. Topology design is beyond the scope of this paper; the interested reader is referred to [6] for a survey on specific network topology design and the routing algorithms designed specifically for them.

The problem of routing network traffic intelligently based on the current network status to meet a number of user defined objectives falls under the class of optimization problems known as the Multi-Commodity Flow Problems (MCFPs). A MCFP is an optimization problem whereby a given set of flows are routed over a given network topology to optimize for a number of objectives while adhering to a set of constraints. Most commonly, solutions to the MCFP are found using Linear Programming (LP). LP solvers are guaranteed to find the optimal solution based on the constraints and conditions set; however, LP solvers are unable to optimize for multiple objectives. Such solvers are also restricted to the use of linear objectives and constraints as otherwise finding a solution becomes an NP-hard problem. As we will see later, the linearity condition, is too restrictive to accurately model some of the network’s behaviour dealt with here. For example, constraining the MCFP such that a flow may only traverse over a single path, is a non-linear constraint and converts the problem to an NP-hard one when solved using LP [7]. When designing routing algorithms, one rarely needs to optimize for a single objective, so that, the MCFP becomes a multi-objective optimization problem and a multi-objective solver is required. Two very common, often conflicting objectives, when dealing with network routing, which are the ones used in this work, are throughput maximization and delay minimization. With a high enough demand, an optimal solution to the MCFP (without the single path constraint), will make use of multipath routing. Multipath routing is key to make the best usage of the available network resources [8, 9].

Multipath routing allows flows to travel over different paths and fall under two categories: Per-Flow Multipath and Per-Packet Multipath. Per-Flow Multipath allows different flows with the same source and destination nodes to take different paths, but a given flow is limited to travel only on a single path. ECMP is an example of a Per-Flow multipath system. In contrast, Per-Packet Multipath allows packets originating from the same flow to take different paths. Per-Packet Multipath provides finer control over the path-flow allocation and thus has the potential to offer improved performance when compared to its Per-Flow counterpart. Even though the finer control of Per-Packet multipath allows for better data rate distribution across multiple paths, to the best of the authors’ knowledge this technique is rarely used in practice due to its negative effects when used over Transmission Control Protocol (TCP) [6]. Given TCP’s prominence, we propose modifications to the Multipath TCP (MPTCP) to overcome this problem.

El-Alfy et al. [10–12] designed a routing algorithm with the aim of minimizing two objectives of an Multi-Protocol Label Switching (MPLS)/Generalised MPLS (GMPLS) network: routing and load balancing costs. Due to the use of multi-objective nature of their algorithm, an alternative to LP was sought with Evolutionary Algorithms (EAs) being the algorithm chosen. The routing cost objective was designed to favour transmission over paths with low cost, while the load balancing objective aims to minimize the use of heavily used links. The routing cost is an abstract metric and can represent the financial cost to use a link or may also be a function of one or multiple link properties such as delay or reliability. A three-dimensional chromosome was designed where each gene is a two-dimensional matrix representing the traffic generated on each link by a given flow. To limit the search space, paths longer than four hops were excluded, and a flow was restricted to a maximum of two paths. The authors noted that, their proposed three-dimensional chromosome design is not very space efficient and its size is dependent on the network size. In contrast, our chromosome size is not directly dependent of the network size, but depends only on the number of flows to route and the set of paths each flow is able to use.

Masri et al. [7] used the Ant Colony Optimisation (ACO) algorithm to solve the MCFP where each flow is restricted to use only one path. This constraint converts the MCFP to an NP-hard problem, which explains the use of the ACO [7]. ACO algorithms are a class of optimizers primarily designed to find the shortest paths within a graph and take inspiration from the behaviour of ants [13]. The ACO is set to minimize both the time required to satisfy all the requests and the network cost. In [14], Evolutionary Algorithms (EAs) were used to find a path that links a source and destination node together with the condition that the path may only pass through a domain once. A domain can be seen as a subnetwork within a larger network. The constraint of passing through a domain only once converts the problem to NP-hard, which is why Evolutionary Algorithms (EAs) are used. The EA designed in [14] only deals with the path finding problem and unlike the work presented here, does not tackle the problem of assigning data rates to flows while taking into account link capacities and other flows using the network. Stefano et al. [15] combined SDN with an Alienated Ant Algorithm (AAA) named A4SDN to optimize for better throughput, delay and packet loss. The AAA is very similar to the ACO algorithm with the exception that ants under the AAA follow the path with the lowest pheromone trail. This modification allows for the generation of solutions with better load balancing performance as the ants do not converge to a single path. By exploiting the network status information offered by SDN and forwarding it to the AAA, A4SDN managed to decrease packet loss by 11% and increase the total network throughput by 16% when compared to the Extended Dijkstra algorithm [16]. A similar concept is used in [17], where an EA is used to find alternative routes to move video streams from congested to less congested paths in the hopes of improving the video stream performance. Initially, all video streams are routed over the shortest path using the Bellman-Ford algorithm. Periodically the SDN controller gathers the network status from the switches and if congestion is detected, the EA algorithm is used to find alternative paths for videos currently transmitted over congested routes. The EA was designed to minimize the path’s aggregate delay and remaining capacity. This technique resulted in a 20% reduction in packet loss and a Peak Signal-to-Noise Ratio (PSNR) improvement of nearly 100% when compared to the Bellman-Ford algorithm.

Not all network optimization approaches use Machine Learning (ML); two particularly interesting non-ML approaches are those by Google [5] and Microsoft [18], which serve as good reference on the practical deployment of global routing solutions on a physical network. In [18] the routing solution is found using LP iteratively on a subset of flows in order of priority, with the objective of maximizing throughput with a preference given to shorter paths. Achieving fairness between flows requires the solution of a number of LP problems, which was considered too costly so was replaced by an approximation. The work in [5] uses similar objectives to those in [18], however the LP solver was replaced by a bespoke greedy heuristic, in the interest of speed. Both [5, 18] use algorithms that are based on LP, optimizing for a single objective. In contrast, in this work multiple objectives are simultaneously optimized. A summary of work closely related to this one has been presented; for an in-depth survey of the various ML algorithms developed for route optimization in SDNs, the reader is referred to [19].

Because of the limitations of LP, including the lack of multi-objective support, Evolutionary Algorithms (EAs) are used in our routing algorithm designs. LP generated solutions, which are optimal for a single objective, are used to compare with the EA generated solutions to gauge the effectiveness of the suboptimal EA algorithm, at least with respect to the objective being optimized by the LP solution. This work focuses solely on the development of the routing algorithm on an already existing network topology and a functioning centralized network architecture is assumed. The routing algorithm proposed here is optimizing for throughput maximization and delay minimization. The EA developed is designed in such a way to allow for easy addition and/or modification of the objectives presented here. The algorithms and results presented in this work assume a static flow set. This is a known limitation, with suggestions for extending our system to work with dynamic flow sets given in the conclusion.

## Routing algorithm design

### Notation

Let G=(V,E) be a loop-free directed graph representing the network topology, where *V* and *E* are the set of nodes and links respectively. Each link is represented by *e* = (*u*, *v*)∈*E* where *u*, *v* ∈ *V* are the link’s source and destination node, respectively. Let $\bar{e}=\left(v,u\right)\in E$ represent the reverse of link *e* = (*u*, *v*)∈*E*. The capacity and cost of each link *e* ∈ *E* is represented by λ_*e*_ and *γ*_*e*_, respectively. The definition of link cost depends on the application, and can take a myriad of values, such as the actual financial cost to use a given link. In this work, the cost of a link is set equal to the link’s delay value. The link’s delay value represents the time taken for information to travel over the said link. Let *F* = {*f*_1_, *f*_2_, …, *f*_*n*_} be the set of *n* flows, where *f*_*i*_ is the *i*th flow in set *F*. Multiple flows can exist between the same source/destination pair. Let *δ*_*i*_ represent the data rate requested by flow *f*_*i*_ ∈ *F*. A path is defined as the sequence of links that connect a sequence of distinct nodes from the flow’s source to the destination. Let *k* represent the maximum number of different paths flows are allowed to take, with the actual number of paths flow *f*_*i*_ is allowed to use given by *k*_*i*_, where *k*_*i*_ ≤ *k*. We define $P_{i}=\left\{p_{i,1},p_{i,2},\ldots ,p_{i,k_{i}}\right\}$ as the set of paths related to flow *f*_*i*_ and $g_{i,j}\in R_{\geq 0}$ as the data rate flow *f*_*i*_ transmits on path *p*_*i*,*j*_. The aggregate delay value of path *p*_*i*,*j*_, denoted by $\phi (p_{i,j})$, is calculated using

$$\begin{array}{c} \phi \left(p_{i,j}\right)=\sum_{e\in p_{i,j}}\;\gamma_{e}\text{.} \end{array}$$ (1)

Finally, let *α*(*g*_*i*,*j*_) represent the TCP acknowledgement flow generated when flow *f*_*i*_ transmits at a data rate of *g*_*i*,*j*_ on path *p*_*i*,*j*_.

### System overview

**Fig 1:**
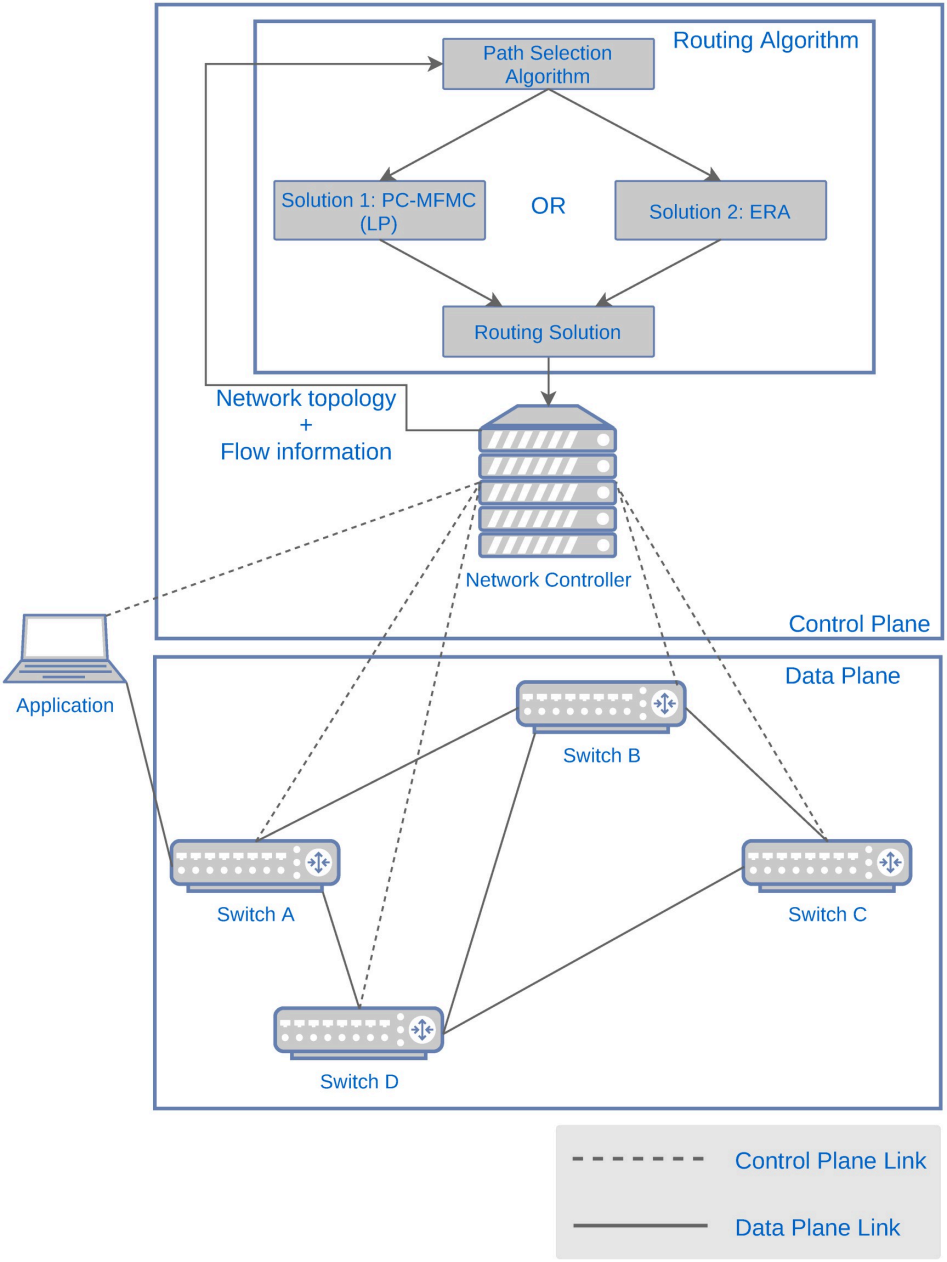
High level overview of the system used.

Fig 1 shows a high level overview of all the modules presented in this work and the links that connect the said modules together. The network is split into two parts; the data and control plane. The data plane is the plane where the actual data packets are transmitted on. The control plane is used for the bidirectional communication between the network controller, network switches and applications. Information shared on the control planes includes application transmission requests, and switch table updates to give a few examples. The network controller has access to both the network topology and its properties, and all the flows that are transmitting, or wish to start transmission over the network. The gathered network status information is fed to the routing algorithm to generate a routing solution. The routing solution contains the paths each flow is allowed to take and the data rate at which to transmit on each path. This work assumes that the network controller has full control over the entire network.

The routing algorithm can be divided into: path selection and data rate optimization. Path selection algorithms generate a set of paths for each flow from a given flow set which is then forwarded to the data rate assignment optimization algorithm. The routing solution is relayed back to the network controller to install the necessary routes on the network switching devices and informs applications requesting transmission permission with the allocated data rate.

### Path selection

The routing algorithms developed in this work rely on external algorithms to supply them with a set of loop free paths that each flow is allowed to use. One of the objectives sought after by all the routing algorithms used here is delay minimization. Therefore, an obvious choice for a path selection algorithm is the *k*-Shortest Path (KSP). The KSP algorithm used here is a variation on Yen’s KSP algorithm [20], where all the paths with an equivalent cost to the *k*^th^ path are chosen at random such that a flow will always have at most *k* paths. This modification is required to have an upper bound on the number of paths available to a flow. Having control over the number of paths is important as it affects the routing algorithm’s complexity.

One shortcoming of the KSP algorithm is the lack of link diversity when used on a highly interconnected network topology, as most of the selected paths will share the vast majority of links between them. From a flow’s perspective, this gives the perceived illusion of being able to transmit over multiple paths; however, allocating data rate to a path reduces the capacity of all the remaining paths that make use of that same link. From the point of view of the routing algorithm, the lack of link diversity limits the different paths a flow may be assigned to. To increase link diversity for a given flow set the *k*-Shortest Edge Disjoint Path (KSEDP) [21] algorithm was considered. Contrary to the KSP algorithm, the paths returned by the KSEDP algorithm do not share any edges, but node sharing is allowed. However, the KSEDP algorithm may be too restrictive in situations where a node is connected to the rest of the network via a single link. To counter this, the *k*-Shortest Relaxed Edge Disjoint Path (KSREDP) algorithm is used instead where the initial path segments that are the only means of communication between a source and destination pair is allowed to be used by multiple paths.

The implementation of both the KSP and KSREDP path selection algorithms described here are based on the algorithm developed by Szcześniak [22]. All the path selection algorithms have their cost set equal to the link’s delay value; the shortest path is equal to the path with the lowest aggregate delay value.

### Multipath transport protocol

The major barrier blocking Per-Packet multipath routing from global adoption is the performance penalty suffered by TCP flows. TCP is the main transport protocol used to date, a system that negatively effects TCP will not find many takers. TCP is a stream oriented transport layer protocol designed for applications seeking a reliable connection between two devices over a computer network. TCP assumes that all packets travel over the same path and builds the congestion control algorithms based on it. This assumption is broken when a flow’s packets are transmitted over multiple paths, as transmitting packets over different paths, with different properties, may lead to packets being received out of order. TCP mistakenly treats this as a sign of congestion and reduces the transmission rate. Due to the exclusive use of Per-Packet multipath by the designed routing algorithms, modifications to the MPTCP are proposed to solve the mentioned problems.

**Background.** mptcp [23] is a transport layer protocol that aggregates multiple TCP sub-flows to improve the flow’s data rate and/or reliability. MPTCP, being a transport layer protocol, does not have the ability to control the path taken by each created TCP sub-flow. Therefore, MPTCP has been originally targeted, and found its first major practical use case in multi-homed devices. Apple first deployed MPTCP with iOS 7 to increase the reliability of the Siri voice assistant [24]. In this case, MPTCP is used to create two connections, one over Wi-Fi and another over LTE for a seamless handover in the event a user loses Wi-Fi connection. The lack of path selection knowledge requires MPTCP to implement a shared congestion control mechanism between all the TCP sub-flows such that multiple MPTCP sub-flows do not starve a single TCP connection from resources if they happen to share the same link [23]. The availability of SDN allows the routing algorithm to gain the intelligence required to distinguish between different MPTCP sub-flows and thus avoid routing them over the same path. Zannettou et al. [25] does just this by exploiting SDN to route MPTCP sub-flows over different paths. However, previous to the work done by Zannettou et al. in [25], the Linux kernel implementation of MPTCP is only able to create one sub-flow for a pair of Internet Protocol (IP) addresses. This limitation has been addressed and fixed by Zannettou et al. [25] and added to version 0.9 of the Linux kernel MPTCP implementation. This change allows MPTCP to open more than one sub-flow for a pair of IP addresses.

**Fig 2:**
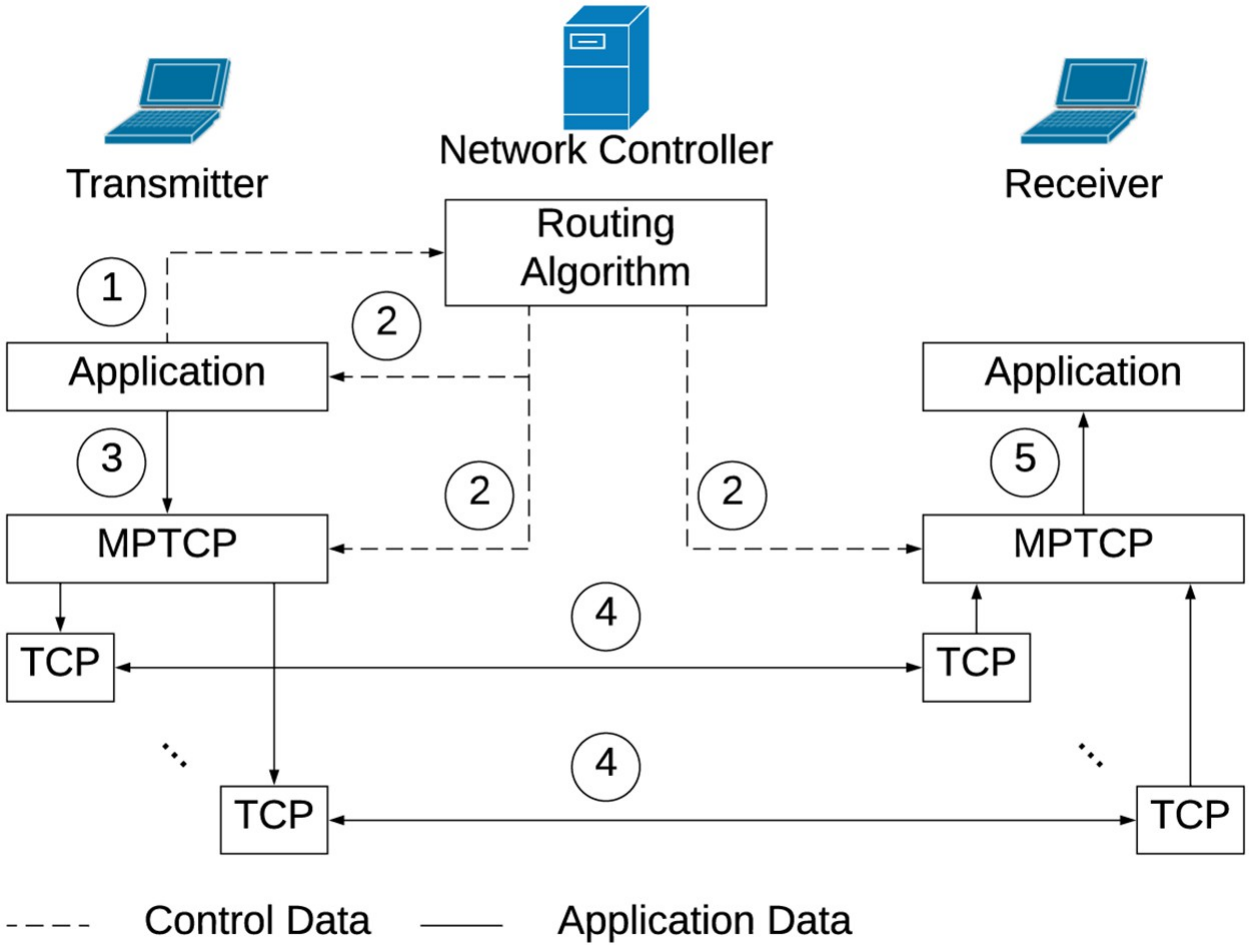
The proposed MPTCP framework. The numbers represent the sequence of events, in order, when an application has data to transmit [27].

**Proposed modifications.** MPTCP does not have the capability of transmitting a sub-flow at a specific data rate out of the box. This functionality is required to adhere with the data rates generated routing solution. The distribution of packets between the different sub-flows (paths) for a given flow is implemented with the help of a stochastic scheduler. A stochastic scheduler is used because of its implementation simplicity and ability to handle any arbitrary split ratio without running into scalability issues. In short, for every packet to be transmitted, a random number is generated for every packet that dictates on which sub-flow the packet will be transmitted on. The size of the bins that each random number falls into is proportional to the data rate assigned to the flow. The proposed MPTCP framework model is shown in Fig 2, which outlines the steps taken in sequence by a flow before starting data transmission over the network. A more detailed explanation of the stochastic splitter and the MPTCP modifications can be found in [26–28], respectively.

### Routing solution 1: Path constrained Max-Flow Min-Cost (LP)

In this work we target the Path Constrained Maximum-Flow Minimum-Cost (PC-MFMC) variant of the MCFP. The PC-MFMC problem is a combination of two problems, solved in succession: Maximum Flow and Minimum Cost. Both problems share the constraint where a flow is restricted to travel on a given path set. Three reasons are behind the use of the path constrained version of the Multi-Commodity Maximum-Flow Minimum-Cost (MMFMC). First, control on the paths a flow is allowed to use allows us to manage the algorithm’s complexity by varying the number of paths each flow is permitted to use. If the algorithm’s complexity is of no concern, flows may be allowed to use all the paths that exist between a source and destination. Second, some flows can be excluded from using certain paths for multiple reasons, including legal ones. Finally, the Evolutionary Routing Algorithm (ERA) routing solution is also path based; therefore, allowing a direct and fair comparison between the two solutions. More information on the PC-MFMC and the non path constrained alternative, referred to as the MMFMC can be found in [29]. The problem formulation of the PC-MFMC follows.

**Maximum flow.** The Maximum Flow problem is solved first and is given by

$$\begin{array}{c} T=\underset{g_{i,j}}{\text{max}}\sum_{i=1}^{n}\sum_{j=1}^{k_{i}}g_{i,j}\text{,} \end{array}$$ (2)

such that

$$\begin{array}{cc} g_{i,j} & \geq 0\;\forall \;i,j\text{,} \end{array}$$ (3)

$$\begin{array}{cc} \sum_{j=1}^{k_{i}}g_{i,j} & \leq \delta_{i}\;\forall \;i\text{,} \end{array}$$ (4)

$$\begin{array}{cc} \sum_{i,j:e\in p_{i,j}}g_{i,j}+\sum_{i,j:\bar{e}\in p_{i,j}}\alpha \left(g_{i,j}\right) & \leq \lambda_{e}\;\forall \;e\text{,} \end{array}$$ (5)

where T represents the total network flow allocated when solving the *Maximum Flow* solution. Constraint Eq (3) ensures that no negative data rate is assigned. Constraint Eq (4) makes sure that no flow is allocated a higher data rate than what it requested. Constraint Eq (5) guarantees that no link is used beyond its capacity, including the acknowledgement flows generated by TCP.

**Minimum cost.** The Minimum Cost solution is formulated as

$$\begin{array}{c} \underset{g_{i,j}}{\text{min}}\sum_{i=1}^{n}\sum_{j=1}^{k_{i}}g_{i,j}\phi \left(p_{i,j}\right)\text{,} \end{array}$$ (6)

such that constraints Eq (3), Eq (4), Eq (5), and

$$\begin{array}{cc} \sum_{i=1}^{n}\sum_{j=1}^{k_{i}}g_{i,j} & =T \end{array}$$ (7)

are met. Constraint Eq (7) guarantees that the *Minimum Cost* solution allocates the same total network flow to that found by the *Maximum Flow* solution. In [29], the *Minimum Cost* solution is set to allocate the same total network flow to that found by the *Maximum Flow* solution by restricting flows to transmit at the data rate allocated by the *Maximum Flow* solution. The Minimum Cost solution given here improves on the one given by Szymanski [29]. The solution in [29] does not have constraint Eq (7) and replaces constraint Eq (4) with

$$\begin{array}{c} \sum_{j=1}^{k_{i}}\;g_{i,j}=D_{i}\;\forall \;i\text{,} \end{array}$$ (8)

where $D_{i}$ represents the data rate allocated to flow *f*_*i*_ by the *Maximum Flow* solution in Eq (2). Compared to the formulations used in [29], the ones presented here do not force the *Minimum Cost* solution to use the flow assignment set by the *Maximum Flow* solution. This gives the *Minimum Cost* solution the freedom to adjust a flow’s allocated data rate in search for a lower cost solution, as long as the same data rate found by the *Maximum Flow* solution is kept. Additionally, the link capacity constraint is updated to take into account the TCP acknowledgement flows. Failing to account for these acknowledgement flows may still provide a routing solution that results in some parts of the network to become congested.

### Routing solution 2: Evolutionary routing algorithm

The objective of the designed ERA is to generate a multi-objective, globally optimized, multipath capable routing solution that maximizes the total network flow and minimizes the application’s mean end-to-end delay. Unlike the LP based solution, the ERA, being a true multi-objective solver, optimizes for all the objectives concurrently. Table 1 summarizes the objectives used by both routing solutions along with an explanation behind their design and use. The ERA presented here is based on [30, 31] by the same authors and uses the NSGA-II algorithm [32]. It shares the chromosome design, crossover operator, total network flow objective, and the excess removal algorithm with the algorithm given in [30]. For completeness, a summary of these components is included in this text.

**Table 1:** Description and relationship between the objectives used by the ERA and PC-MFMC problem solved using LP. ✔: Objective present in algorithm. ✗: Objective not present in algorithm.

Objective	Algorithm		Comments
	ERA	PCMFMC	
Flow Maximization	✔	✔	Aims at maximizing the total flow passing through the network at any given time.
Cost Minimization	✗	✔	Aims at minimizing the total network cost. This translated to delay minimization because the cost of a link is set equal to its delay value.
Estimated Mean End-to-End delay	✔	✗	Aims at minimizing the end-to-end delay experienced by a flow. This is a more accurate representation of reality than the cost minimization objective defined in the above row. However, this objective is non-linear; therefore, cannot be used with LP.

#### Chromosome design

**Fig 3:**
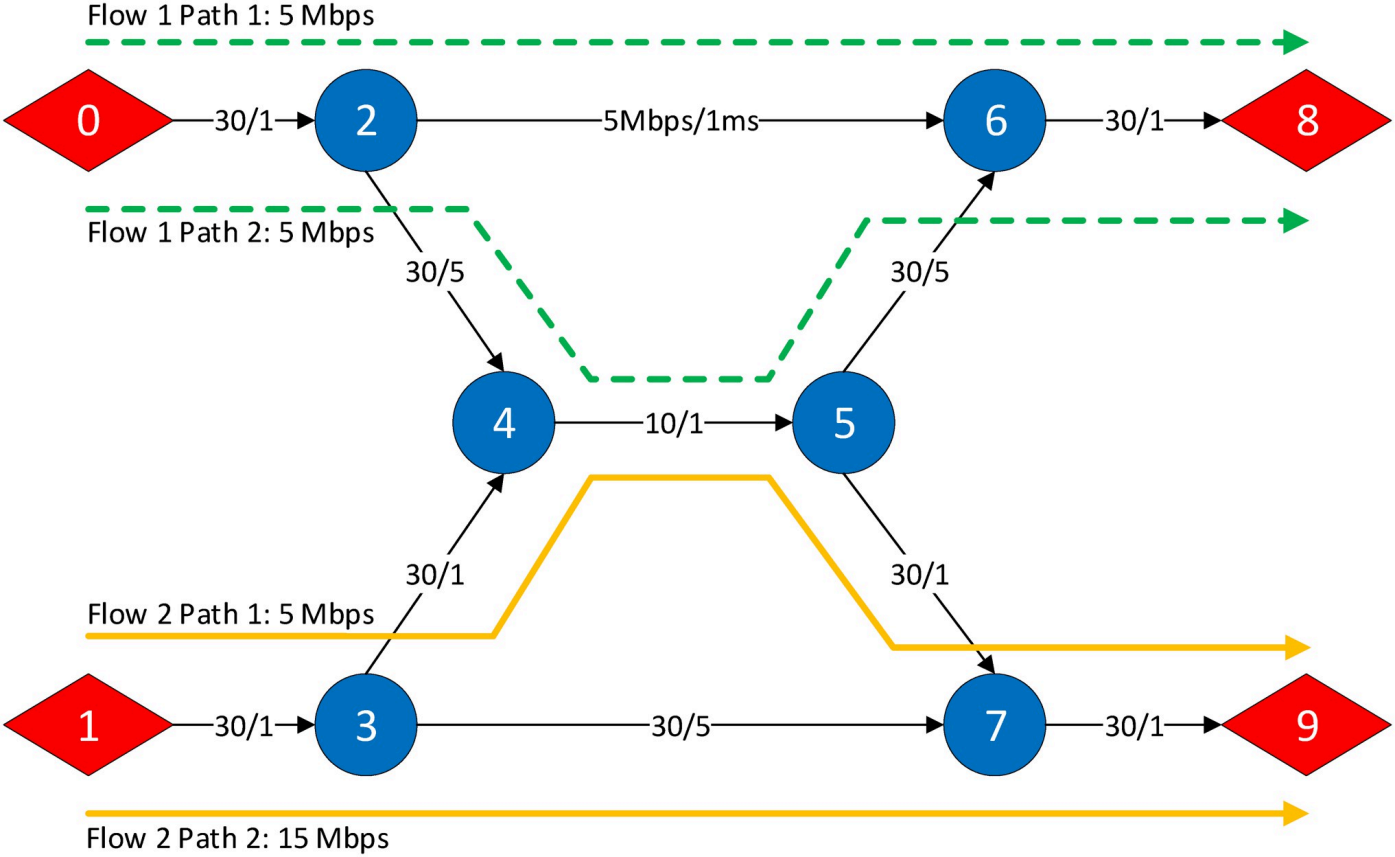
Butterfly network used to explain the chromosome representation of a network. Flows 1 and 2 are transmitting at a data rate of 10 Mbps and 20 Mbps respectively. Each flow is allowed to transmit on two paths, as shown by the green and yellow path markers [30].

The design of the chromosome is the foundation of any EA as it represents the way a solution is formulated. The chromosome *C* is defined as the sequence *C* = (*G*_1_, *G*_2_, …, *G*_*n*_) where $G_{i}=\left(g_{i,1},g_{i,2},\ldots ,g_{i,k_{i}}\right)$ is the sequence of genes related to flow *f*_*i*_. The chromosome has been carefully designed to accurately represent a routing solution, include the flow conservation constraint and scale independently of the underlying network topology. The flow conservation constraint ensures that all data transmitted from a source node must reach its destination node in its entirety with no losses incurred at the relay nodes. As each gene in the chromosome represents the data rate to transmit on a path, as long as the path starts from the source and ends at the destination node, then the flow conservation constraint is implied. Using the set-up shown in Fig 3 as an example, where Flow 1 is transmitting at 10 Mbps, Flow 2 is transmitting at 20 Mbps and both have *k* = 2, the chromosome representation is equal to *C* = ((5, 5), (5, 15)).

#### Objective functions

The fitness of a routing solution is based on two objectives: the total network flow, and the application’s estimated mean end-to-end delay, represented by $O_{1}$, and $O_{2}$, respectively. Throughput maximization and delay minimization are the objectives chosen in this work as they are two of the most common and generally applicable metrics used when judging the performance of a given network. Having said this, routing algorithms that require the optimization of a highly specific network metric for which the objectives presented here are either too generic, or do not fully encompass the requirements can either add new objectives, or modify the existing ones.

**Total network flow maximization.** One of the fundamental requirements of a routing algorithm is to maximize the total network data rate, as this has a direct impact on the network efficiency and flow satisfaction rate. The total network flow objective is given by

$$\begin{array}{c} O_{1}=\sum_{i=1}^{n}\sum_{j=1}^{k_{i}}g_{i,j}\text{.} \end{array}$$ (9)

This objective is normalized by dividing it with the total requested data rate across all flows, $\sum_{i=1}^{n}\delta_{i}$.

**Estimated mean end-to-end delay minimization.** When using the modified MPTCP protocol, the mean delay experienced by the application is affected by both the path delay values and the data rate transmitted on each path. An application’s end-to-end delay is defined as the time taken from when the transmitting application sends a byte of data, to when the receiving application receives that same byte of data. Modelling the interaction between the packets transmitted on different paths to calculate the mean end-to-end delay is not trivial. Using a simple queue model, we observed that the average end-to-end delay tends to be very close to the largest path delay value from the set of paths used. We simplify the application’s end-to-end delay measurement by setting it equal to the largest delay value from the set of paths used by a given flow. Based on this simplified model and the goal of minimizing the delay experienced by a flow; this objective minimizes the transmission rate on paths with large delay values from the set of paths available to a flow. The objective’s formulation is given by

$$\begin{array}{c} O_{2}=\sum_{i=1}^{n}F_{i}\text{,} \end{array}$$ (10)

where

$$\begin{array}{c} F_{i}=\frac{\sum_{j=1}^{k_{i}}g_{i,j}}{\sum_{i=1}^{n}\sum_{j=1}^{k_{i}}g_{i,j}}\times \text{max}\left(\phi \left({\hat{P}}_{i}\right)\right)\text{.} \end{array}$$ (11)

$F_{i}$ represents the objective value for flow *f*_*i*_ and the set ${\hat{P}}_{i}\subseteq P_{i}$ includes all the paths *p*_*i*,*j*_, where *g*_*i*,*j*_ > 0. $F_{i}$ is normalized with respect to the total flow rate that is allocated for that given solution such that the final value is independent of the solution’s total network flow value. Note that this objective is non-linear because the flow’s delay value is conditionally based on which paths the flow is currently using; thus, it cannot be used with an LP solver. This objective is normalized by dividing it with the cost of the path with the largest delay from the set of all paths.

#### Crossover

**Fig 4:**
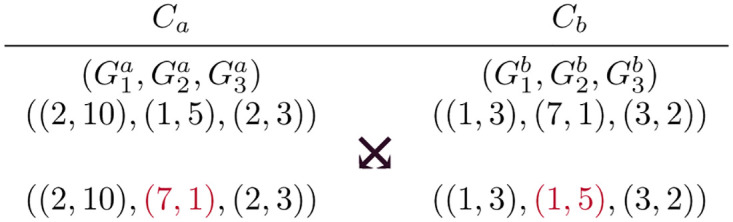
Crossover example where the genes related to Flow 2 are swapped to generate a new routing solution.

The crossover operator is used to generate new offspring (routing solutions) by mating two chromosomes together, referred to as the parent chromosomes, to generate two new offspring solutions. Two parent chromosomes, *C*_*a*_ and *C*_*b*_, are selected using dominance based tournament selection [33]. For every crossover operation, a mixing ratio z∈U(0,1) is chosen. U(0,1) represents a random source uniformly distributed between 0 and 1. Each gene in the sequence $C_{a}=\left(G_{1}^{a},G_{2}^{a},\ldots ,G_{n}^{a}\right)$ is swapped with its corresponding sequence $C_{b}=\left(G_{1}^{b},G_{2}^{b},\ldots ,G_{n}^{b}\right)$ with probability z∈U(0,1). A random mixing ratio is used to allow the possibility of an offspring to inherit most of the genes from a single parent. Fig 4 shows an example of a crossover operation where the genes related to Flow 2 are swapped to create two new routing solutions.

#### Mutation

While the crossover operator generates new routing solutions by combining chromosomes together, it does not modify any of the flow’s data rate assignments as this task is left to the mutation operator. The mutation operator works on a single chromosome, modifying the gene sequences related to a fraction *μ* of flows, chosen at random, within that chromosome. For every gene sequence *G*_*i*_ that is selected for mutation, the mutation operation selects a subset ${\tilde{P}}_{i}\subseteq P_{i}$ of paths which flow *f*_*i*_ is allowed to use. Once ${\tilde{P}}_{i}$ is chosen, the paths are considered in random order, transmitting as much data as possible until the flow’s requested data rate is met, or all paths are used. This data rate assignment does not exceed any link capacity and takes into account all the other flow data rate assignments. The path set ${\tilde{P}}_{i}$ is generated using one of the two methods explained next. Method selection is done at random with both methods having equal probability.

**Minimize the maximum path delay.** This method attempts to minimize the probability of including paths with high delay values from the set of paths a flow is allowed to use. For a given flow *f*_*i*_, all paths with $\frac{\phi (p_{i,\text{min}})}{\phi (p_{i,j})}\geq z$ are included in ${\tilde{P}}_{i}$, where z∈U(0,1) and *p*_*i*,min_ represents the path with the lowest delay from the set *P*_*i*_, given by

$$\begin{array}{c} p_{i,\text{min}}=\underset{p_{i,j}\in P_{i}}{\text{arg min}}\phi \left(p_{i,j}\right)\text{.} \end{array}$$ (12)

Paths with a higher delay value have a diminishing probability of being selected as this has a direct impact on the flow’s end-to-end delay performance.

**Maximize flow.** The objective of this method is to transmit as much of the flow’s requested data rate as possible over all available paths; thus, ${\tilde{P}}_{i}=P_{i}$.

#### Initial population generation

The initial population is generated using the following procedure. For each flow *f*_*i*_, the number of paths the flow is allowed to use, *ν*, is randomly selected using a uniform distribution from the set *ν* ∈ {0, 1, 2, …, *k*_*i*_}. Subsequently, *ν* paths are chosen at random from the set *P*_*i*_. The fraction of data rate the flow is to transmit compared to its requested data rate *δ*_*i*_ is randomly determined using

$$\begin{array}{c} {\hat{\delta }}_{i}=\delta_{i}\times z\text{,} \end{array}$$ (13)

where z∈U(0,1). For each of the chosen paths *p*_*i*,*j*_, the smallest link capacity along that path is calculated by

$$\begin{array}{c} \rho \left(p_{i,j}\right)=\underset{e\in p_{i,j}}{\text{min}}\;\lambda_{e}\text{,} \end{array}$$ (14)

and the corresponding gene is set to $g_{i,j}=\text{min}\left(\rho \left(p_{i,j}\right),{\hat{\delta }}_{i}\right)$. Genes for paths that were not in the chosen subset are set to zero. This population initialization method may create solutions that break the constraints defined by the MCFP. In such instances, the chromosome is repaired using the methods described in the following section.

#### Constraint handling

The chromosome’s design already ensures that a number of constraints are met. Two additional constraints that are not implicitly satisfied remain and are: the flow over-provision constraint and the link capacity constraint. Chromosomes are first checked for over-provisioned flows, followed by a check for over-capacity links. The order is important because it is pointless to fix over-capacity links when over-provisioned flows may still be present in a given solution. After a crossover operation is performed, the two newly generated solutions are checked against the link capacity constraint. The crossover operation does not modify the flows’ data rate assignment; therefore, there is no need to validate the flows’ over-provision constraint. The mutation operation does not require any validation because the current network usage is taken into account when assigning data rate on paths, and no flow is assigned more data rate than requested. Finally, the method used to generate the initial population requires that each chromosome is checked for both the flow over-provision and link capacity constraints. Excess flow is removed using the excess removal algorithm explained below from *G*_*i*_. For every link found to be over capacity, the excess removal algorithm explained below is used to remove the excess in an unbiased way from the genes in the set {*g*_*i*,*j*_: *e* ∈ *p*_*i*,*j*_}. Since the excess removal operation affects a whole path, links other than the one that triggered the operation may be affected. To reduce bias, links are considered and repaired in a random order. This process is terminated when no over-capacity links remain. For a detailed explanation, refer to [28, 30].

#### Excess removal algorithm

The excess removal algorithm is used to remove a known excess amount from a set of values while being as fair as possible in relation to the amount to remove from each element in the given set. Let *G* = {*g*_1_, *g*_2_, …, *g*_*κ*_} represent the sequence of *κ* genes, determined by the flow over-provision constraint or link capacity constraint, from which we need to remove an excess value of *τ*. That is, we want to determine an updated sequence of genes $G^{\prime }=\left\{g_{1}^{\prime },g_{2}^{\prime },\ldots ,g_{\kappa }^{\prime }\right\}$ such that $0\leq g_{i}^{\prime }\leq g_{i}$, *i* ∈ {1, 2, …, *κ*} and $\sum_{i=1}^{\kappa }g_{i}-g_{i}^{\prime }=\tau$. Let *ξ*_*i*_ represent the amount to remove from gene *g*_*i*_, such that $g_{i}^{\prime }=g_{i}-\xi_{i}$. We randomly determine each *ξ*_*i*_ with the constraints imposed by *G*, *τ*, and previously determined *ξ*_*j*_, *j* < *i*:

$$\begin{array}{cc} 0\leq & \xi_{i}\leq g_{i} \end{array}$$ (15)

$$\begin{array}{cc} \tau -\sum_{j=1}^{i-1}\xi_{j}-\sum_{j=i+1}^{\kappa }g_{j}\leq & \xi_{i}\leq \tau -\sum_{j=1}^{i-1}\xi_{j} \end{array}$$ (16)

Each *ξ*_*i*_ is chosen uniformly at random within a range satisfying both constraints. To avoid introducing a bias in the evolutionary algorithm, the genes in *G* are considered in a random order.

### Complexity analysis

#### ERA

Let *ϵ* represent the number of links in a given graph, where *e*_*i*_ ∈ *E* represent the *i*^th^ link. Let *θ* represent the total number of paths (chromosome size), given by

$$\begin{array}{c} \theta =\sum_{i=1}^{n}\left|P_{i}\right|\text{,} \end{array}$$ (17)

where |*P*_*i*_| = *k*_*i*_ represents the cardinality of the set *P*_*i*_. Let *M* and P represent the number of objectives and population size, respectively. The complexity of each function used by the ERA is given in Table 2 where functions have been reduced to their most dominant function and constants removed. Let *χ* represent the number of generations, *ω* the crossover probability, and *ψ* the mutation probability. Using the equations in Table 2, and assuming the worst case scenario where *ω* = *ψ* = *μ* = 1, the complexity of the developed ERA is equal to

$$\begin{array}{c} O(P\left(\theta \epsilon^{2}+nk\right)+\chi \left(\theta \epsilon^{2}+n\theta \epsilon +nk+P\theta +P^{2}\right))\text{.} \end{array}$$ (18)

**Table 2:** Time complexity of each ERA function.

Function	Complexity
Flow Over-Provision Constraint	*O*(*nk*)
Link Over-Provision Constraint	*O*(*θϵ*^2^)
Initial Population Generation	$O(P\theta {∊}^{2}+Pnk)$
Crossover	*O*(*n*+ *θϵ*^2^)
Mutation	*O*(*μn*(*θϵ*+ *k*))
NSGA-II Selection [32]	$O(MP^{2})$
Total Network Flow Objective ($O_{1}$)	*O*(*θ*)
Estimated Mean End-To-End Delay ($O_{2}$)	*O*(*θ*)

The derivations for the complexity equations in Table 2 are given in [28].

#### LP

**Fig 5:**
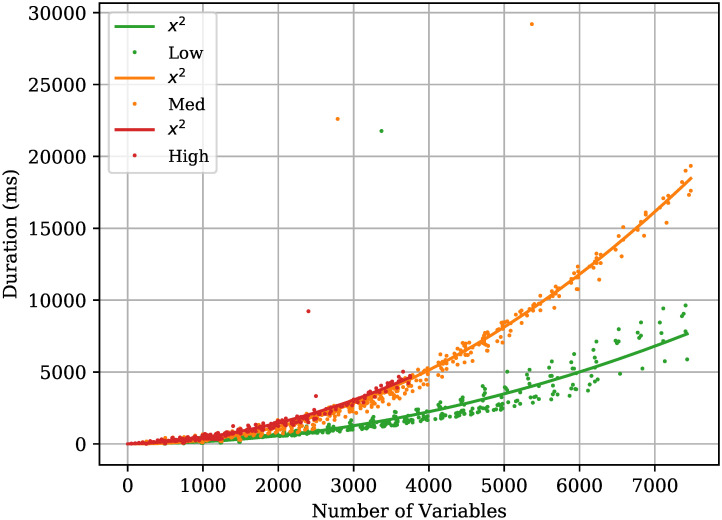
Time needed for LP to solve the PC-MFMC. Time taken for the LP algorithm to find a solution to the PC-MFMC problem as the number of variables is increased, grouped by the network load. *x*^2^ plot refers to the degree 2 polynomial generated when fitting a curve to the generated data points. The Low, Med, High legend labels refer to the different network loads used.

The GNU Linear Programming Kit (GLPK) library does not provide information on the complexity and scalability of the algorithms used to solve LP formulations. Therefore, empirical evidence is used to determine the scalability of the developed LP routing algorithm that solves the PC-MFMC problem. Fig 5 shows the time taken by the LP solver to find a solution to the PC-MFMC problem as the number of variables increases, grouped by the network load. In this context, variables refer to all the *g*_*i*,*j*_ variables that the LP solver needs to find a suitable value for. Using curve fitting, it is clear that in general, the LP solver used in this work scales quadratically with the number of variables. Note that there are particular instances where the time required to find a solution is more than double the time required by solutions with the same number of variables. The reason behind these outlier values needs to be investigated further. To the best of the authors’ knowledge, the most recent work on how to solve LP problems efficiently is the one by Cohen et al. [34], which still scales quadratically with the number of variables.

#### ERA vs LP

Solving the PC-MFMC problem using LP scales quadratically with the number of variables. The number of variables is equivalent to the total number of paths in a given solution, which is equal to the chromosome size (*θ*). On the other hand, the developed ERA scales quadratically with the number of links in a given topology (*ϵ*) and the population size (P). Assuming that the ERA is used over a fixed network topology and the population size is kept constant, the designed ERA scales linearly with the chromosome size which is an improvement over the LP solution. The ERA proposed here makes use of non-linear objectives as they offer a more accurate representation of the network behaviour compared to their linear counterparts. Even though the ERA is inherently suboptimal, when the system to be modelled has non-linear properties, as is this case in this work, alternate solvers to LP need to be sought. Finding a solution to a non-linear problem using LP can convert the problem to an NP-Hard one [7].

## Experimental setup

### Network topology

The 2017 GÉANT network topology is used as it models an actual network topology and has a dense network core with multiple paths between any two locations. Such a feature is important to this work as it allows us to test the multipath capability of our routing algorithms. Detailed information on the topology, link capacity and delay values used can be found in [28, 31].

### Flow setup

In this work we simplify the problem by using a static flow set, meaning that no flows enter or exit the network for the duration of the simulation. Three network loads are used: Low, Medium and High. Except for the high network load, that goes up to 150 flows, the number of flows ranges from 50 to 300 in steps of 50. The high network load scenario is capped at 150 flows, because network capacity is already exceeded at this point, with only 70% of the total requested flow rate being allocated. Five flow sets are generated for each network load. The flow data rate is generated using a normal distribution with mean (standard deviation) for the low and high network set at 5Mbps(0.25Mbps) and 25Mbps(2.5Mbps), respectively. The flow data rate values for the low and high network load setup are chosen to represent Definition (HD) and Ultra High Definition (UHD) video transmission, respectively. The medium load setup has an equal number of flows having a low and high network load profile. Under all scenarios considered here, the flows’ source and destination nodes are selected randomly with the selection probability directly proportional to the node’s total outgoing or incoming capacity, respectively. Flows with identical source and destination nodes are not allowed.

### Solver frameworks

Solutions to the LP formulations are found using the GLPK [35] library accessed through the LEMON’s [36] library interface. LEMON’s GLPK interface has been updated to run the glp_exact function after running the glp_simplex function to improve numerical stability. The use of the glp_exact is required as otherwise, flows may be allocated very small negative data rates, even though a constraint is set where solutions are only allowed to use positive numbers. The ERA designed in this work is implemented using the Distributed Evolutionary Algorithms in Python (DEAP) v1.3 [37] library.

### Network simulations

Network simulations are carried out using the Network Simulator version 3.29 (ns-3) [38]. Custom devices are developed to simulate the required functionality of an SDN switch and the Per-Packet Flow Splitting (PPFS) switch developed in [26]. All switches are assumed to have unlimited buffers to eliminate the effect of packet loss caused by buffer overflow. Although this is unrealistic, this assumption simplifies the analysis of the network performance results. All flows are assumed to transmit at a Constant Bit Rate (CBR) with a data packet size of 590 bytes including all the necessary headers with each TCP acknowledgement packet being 54 bytes long. Methods of how to shape bursty traffic to have a profile similar to a CBR exist, with Szymanski [29] presenting one such method. Using the above packet sizes, and the assumption that TCP transmits an acknowledgement packet for every two data packets received [39], the TCP’s acknowledgement rate *α*(*g*_*i*,*j*_) = 0.0458 × *g*_*i*,*j*_. The *NewReno* tcp congestion control mechanism is used [40]. Except for Open Shortest Path First (OSPF), applications transmit at the rate assigned by the routing algorithm, not that requested. Data rate transmission modification is possible as SDN allows for bidirectional communication between the routing algorithm hosted on the network controller and the application. OSPF lacks such functionality; thus, OSPF results are generated by setting the flows to transmit at their requested data rate. For each TCP connection/sub-flow created by the MPTCP protocol, the TCP transmit and receive buffer size is automatically adjusted such that it is large enough to support transmitting at the data rate assigned by the routing algorithm on that given path. The buffer size in bytes is calculated using the bandwidth delay product [39] as given by

$$\begin{array}{c} \text{Buffer}\;\text{Size}=\text{min}(\frac{g_{i,j}\times \text{RTT}}{8},4096)\text{,} \end{array}$$ (19)

where Round Trip Time (RTT) is given in seconds and *g*_*i*,*j*_ is in bits per second. A minimum buffer size of 4096 bytes is set to match the value used by the TCP implementation in the Linux kernel. Under all scenarios presented here, the routing tables are populated before packet transmission starts, eliminating any routing protocol overhead.

Due to the lack of a native ns-3 MPTCP protocol implementation, the TCP sub-flow generator, and scheduler were developed as these two blocks are sufficient to test the performance of the updated MPTCP protocol. The TCP sub-flow generator is the module that creates a number of TCP sessions, where the number of sessions to open, and which port numbers to use on each session is specified by the routing algorithm. The developed MPTCP stochastic scheduler distributes packets between the different TCP sessions (each TCP session is equivalent to a path), where the split ratios are given by the routing algorithm. Due to time constraints, the shared congestion control algorithm is not implemented and the MPTCP receiver applications are assumed to have infinite receiver buffers to avoid the need to implement MPTCP’s acknowledgement mechanism to recover from packet losses caused by receiver buffer overflow. We do not see any reason why the shared congestion control used by MPTCP should negatively impact performance due to the use of a globally optimized routing solution. Packet loss and congestion caused by the dynamic network environment are handled by the underlying TCP sub-flows and are handled by our developed MPTCP model.

Network simulation results were generated by allowing the simulation to run for 120 simulation time seconds.

## Results

In the results that follow, *Goodput* is defined as the rate at which an application is able to generate or consume data. *Delay* is defined as the time taken from when the transmitting application sends a byte of data, to when the receiving application receives that same byte of data. Any time used waiting to deliver a block of data to the application in its correct order is included in the delay measurements. This setup is used to accurately represent the performance of an application using the protocols under test.

### EA parameter selection

Finding the optimal parameter values for an EA is itself a multi-objective optimization problem, and depends heavily on the algorithm’s use case. Although several tests were carried out to ensure that the values chosen work well for a wide number of cases, as is demonstrated by the results presented in this work, we do not state that these are the optimal values. The EA parameter values need to be set up in such a way to strike a balance between the rate of convergence and the even coverage of the optimal Pareto Front. On the one hand, the improvement between generations should not be minute, as this would require a very large number of generations before reaching a good enough approximation of the true Pareto Front. And on the other, there should not be a huge gap between the current and previous population as this may skip over solutions that make up the true Pareto Front. In addition, the solutions found by an EA, need to be evenly spread over the entire Pareto Front. Having a number of solutions either grouped in a relatively small area, or heavily biased to one objective, is a sign that the EA is not exploring all areas equally. The ideal set of EA parameters needs to offer a steady and gradual improvement with each generation until a satisfactory Pareto Front is generated. The EA parameter values are highly dependent on the design and function of the crossover and mutation operators. Due to the wide selection of such operators, only recommendations on what values to use exist with most EA implementations favouring a high crossover and low mutation rate. The EA parameters used here are given in Table 3. In this work, the crossover probability (*ω*) is set to 0.9, as such value is commonly used with the NSGA-II algorithm [32] and has been found to work well in our setup. During the EA parameter selection process, the effect on the EA’s evolution progress when changing a single parameter value has been thoroughly analysed and tested before taking the final decision. An in-depth explanation of how each value was chosen is given in [28].

**Table 3:** EA parameters.

Parameter	Value
Population Size (P)	800
Number of Generations (*χ*)	400
Crossover Probability (*ω*)	0.9
Mutation Probability (*ψ*)	0.2
Fraction of flows to be mutated (*μ*)	0.2

### Choosing *k*

**Fig 6:**
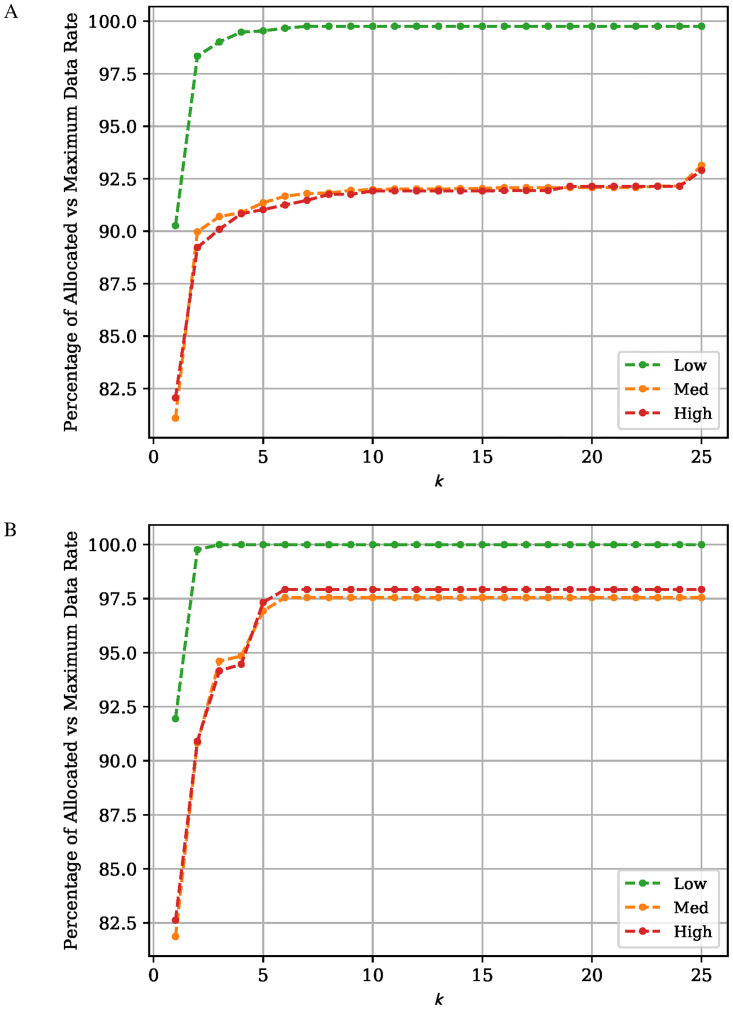
Allocated data rate for constrained vs unconstrained MFMC. Percentage of the Allocated Data Rate when solving the PC-MFMC problem compared to the unconstrained Maximum Flow problem, with varying *k*. Solutions to both problems is found using LP. All five flow sets for a given network load and the highest number of flows for a given network load are used; 300 Flows for the Low and Medium network flow, and 150 flows for the High network load. A: Path Selection Algorithm: KSP. B: Path Selection Algorithm: KSREDP.

Two different path selection algorithms: KSP and KSREDP are presented in this work. Both algorithms take a single parameter, *k*, which represents the maximum number of paths a flow is allowed to take. When choosing a value for *k* a compromise needs to be reached between the algorithm’s running time and path variety. To choose *k*, the total assigned network flow when solving the unconstrained Maximum Flow problem is compared with the solution of the PC-MFMC problem. The solutions to the two aforementioned problems is found using LP because of LP’s optimality guarantee, meaning that any performance difference is solely attributed to the change in *k* value. The unconstrained Maximum Flow problem can be seen as the PC-MFMC problem where *k* = ∞. For the formulation of the unconstrained Maximum Flow problem, the reader is referred to Eq (11) in Section III of [31]. Fig 6 shows the total network flow allocated by the PC-MFMC problem as a fraction of that found by the unconstrained version. The results in Fig 6, confirm that the KSREDP algorithm is able to find solutions with a higher total network flow for a smaller *k* when compared to the KSP algorithm. The KSREDP is more suited for scenarios that prioritize throughput over delay as it may have better performance in terms of throughput. However, this comes at the cost of increased risk of worse delay performance, where the KSREDP algorithm can match the delay performance of the KSP algorithm, but never surpass it. On the contrary, the KSREDP solutions are more likely to offer worse delay performance when compared to KSP. Using Fig 6 as a reference, there is marginal difference in the allocated network rate between *k* = 5 and *k* > 5; therefore, in this work we set *k* = 5. Reaching the same network allocation rate as the unconstrained Maximum Flow when using the PC-MFMC problem is possible; however, this would require a large *k* value that would increase the complexity in such a way that would make running and testing the ERA infeasible.

### Routing algorithm performance

#### Data rate allocation

**Fig 7:**
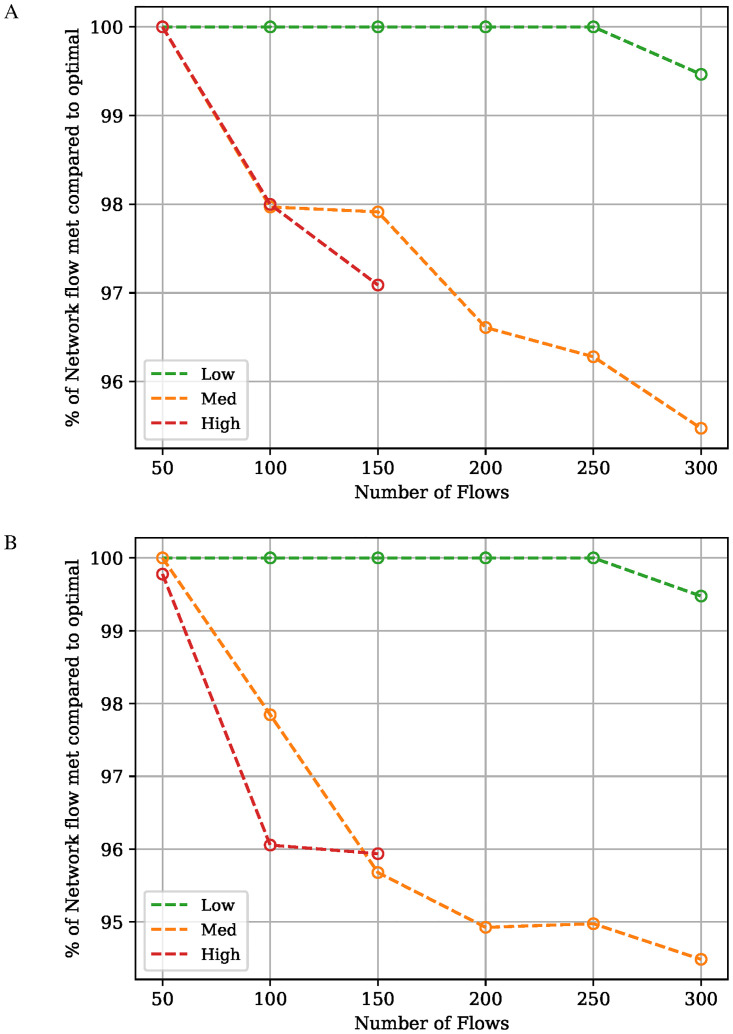
Comparison of demand reached between ERA and PC-MFMC. Plot illustrating the Percentage of demand achieved by the ERA algorithms when compared to the optimal solution to the PC-MFMC problem solved using LP. The solution with the largest network flow found by the ERA is used. A: Path Selection Algorithm: KSP. B: Path Selection Algorithm: KSREDP.

The performance of the developed ERA is compared with the optimal solution provided by LP. The solutions generated by LP for the PC-MFMC problem are only truly optimal for a single objective; in our case, the maximization of the total network flow. For a fair comparison between the algorithms, the highest network flow solution found by the ERA is compared with that found by LP, for all the flow sets and network loads used here. Fig 7 shows the percentage of demand the ERA managed to satisfy when compared to the LP found solution. From the results, we can observe that the solutions found by the ERA match those found by LP very closely for a lightly loaded network. As the network load and number of flows increase, the difference in the satisfied demand between the solutions found by the ERA and LP increases by at most 7% and 8% when using the KSP and KSREDP algorithms, respectively. These results serve to show that even though the ERA is suboptimal, it manages to satisfy, on average, 98% of the demand reached by the optimal solution.

#### Hybrid

**Fig 8:**
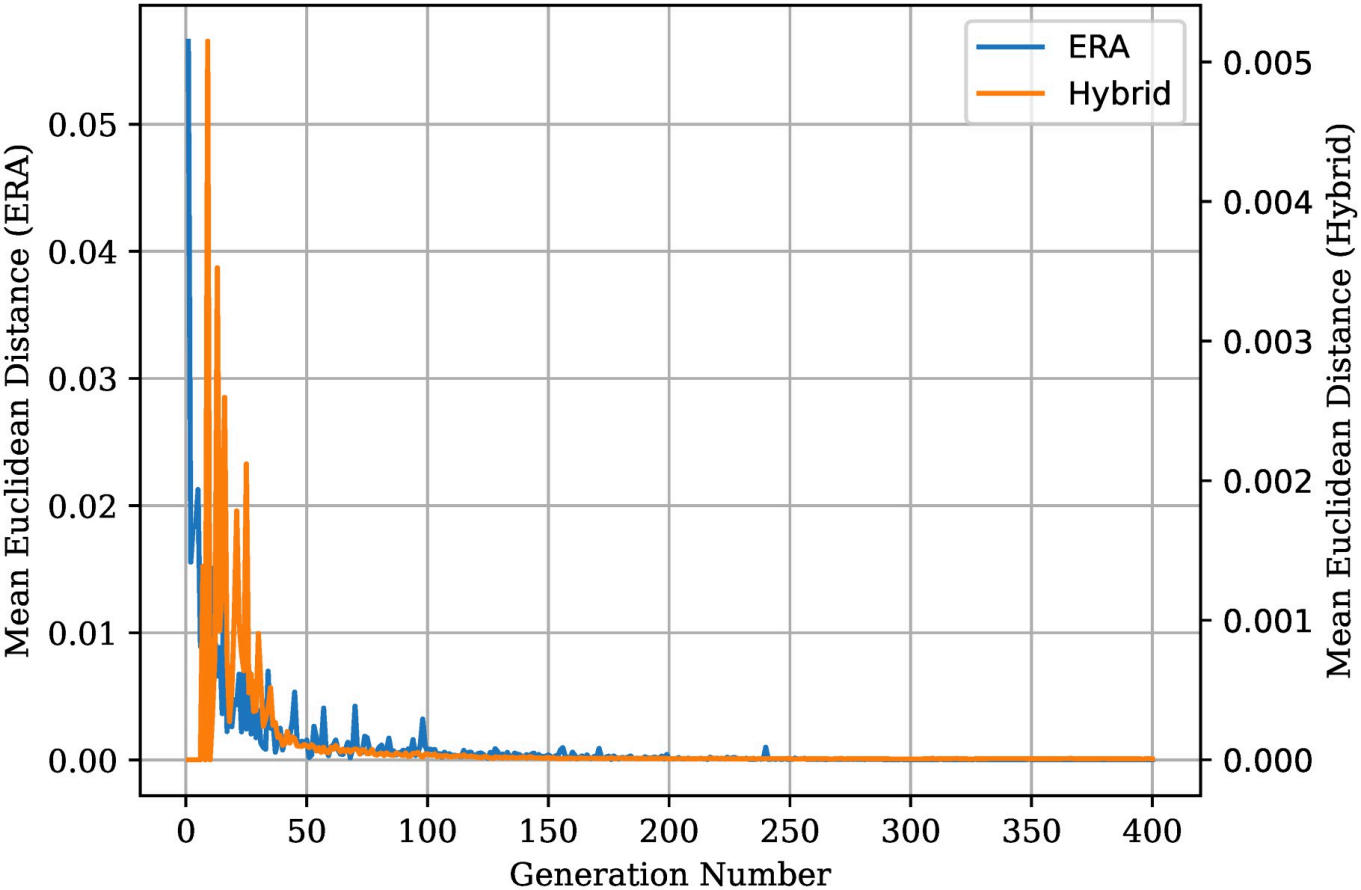
Mean Euclidean distance between successive Pareto Fronts for both the Hybrid and non-Hybrid versions of the ERA.

The Hybrid algorithm is a minor modification over the developed ERA, where the LP optimal solution is added to the initial population. Note that the population size remains equal to 800 including the LP optimal solution. There are three main advantages of this inclusion. First, thanks to the elitist nature of the NSGA-II algorithm, the Pareto Front generated by the Hybrid ERA is guaranteed to contain the LP found solution, or an alternative that has the same allocated total network flow but is better in any of the other objectives. Second, the Hybrid routing algorithm is able to provide multiple valid solutions. Having multiple valid solutions at the end of a run is beneficial as it allows the algorithm to explore the search space for all objectives without any bias towards any objective. Choosing a solution from the generated pool of solutions means that a compromise between the objectives has to be made; however, this bias between objectives was made when taking into account the entire pool of generated solutions. This is very different from setting biases on the objectives before the algorithm even starts, which requires very deep knowledge of the area to get such biases right. More information on this can be found in [28, 33] with examples of how the provision of multiple results is beneficial in a network setting can be found in [30]. Third, the convergence rate is improved as shown in Fig 8 where the mean Euclidean distance for the Hybrid and non-Hybrid version are compared. The mean Euclidean distance, *M*_*A*,*B*_, between the set of solutions on the Pareto Front in generations *A* and *B*, respectively *S*_*A*_ and *S*_*B*_, is given by

$$\begin{array}{c} M_{A,B}=\frac{1}{|S_{A}|}\sum_{a\in S_{A}}\underset{b\in S_{B}}{\text{min}}d\left(a,b\right)\text{.} \end{array}$$ (20)

**Fig 9:**
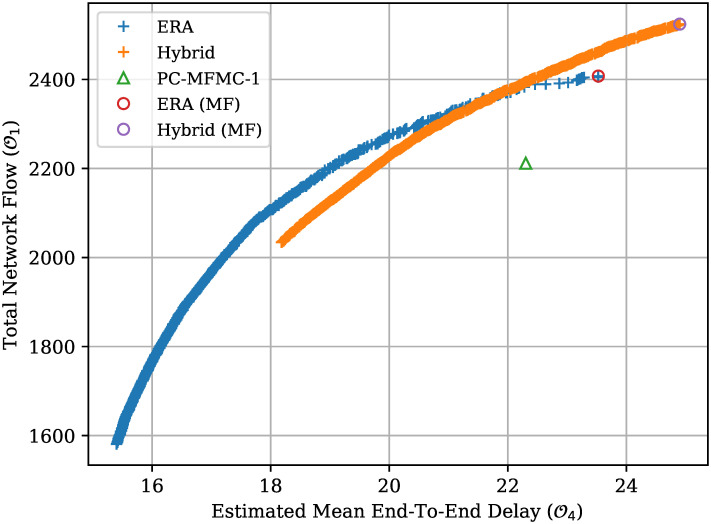
Hybrid vs Non-Hybrid Pareto Front. ERA represents the Pareto Front generated by ERA when the population is generated entirely at random. Hybrid represents the ERA and LP hybrid routing algorithm. PC-MFMC-1 represents the solution found by LP when only the shortest path is available and is used to represent the maximum attainable network flow by OSPF. MF marks the solution with the largest Network Flow value.

*d*(*a*, *b*) refers the Euclidean distance between *a* and *b*, where *a* and *b* are tuples of the normalized objectives for the algorithm used. The Hybrid algorithm reports a mean euclidean distance of zero for the first few generations because the PC-MFMC solution dominates all the generated solutions. The obvious downside of such a Hybrid algorithm is that both the LP and EA algorithms have to be run. Fig 9 shows the generated Pareto Front for both the standard ERA and Hybrid routing algorithm. From Fig 9 it can be observed that the Hybrid algorithm found a number of solutions that dominate those found by the standard ERA. However, the final Pareto Front of the Hybrid algorithm is much tighter than the one found by the non-Hybrid version. This is not ideal, as it shows that the Hybrid algorithm is not looking into all directions equally, but rather favouring solutions with a high network flow. The Hybrid algorithm was developed and tested during the last stages of this research; therefore, there was not enough time left to identify the cause of such a constricted Pareto Front. To try and identify the cause of such a problem, one may start by looking at the entire evolution of the algorithm to determine at which stages the Pareto Front starts to close up. If this behaviour is noticed even at the early stages of evolution, the insertion of the LP optimal solution in the first generation might be premature. Having an optimal solution in the initial population might require tweaks to the ERA parameters to either increase or decrease the aggressiveness of the mutation operator.

### Simulated network performance

#### ERA vs Hybrid vs OSPF

**Fig 10:**
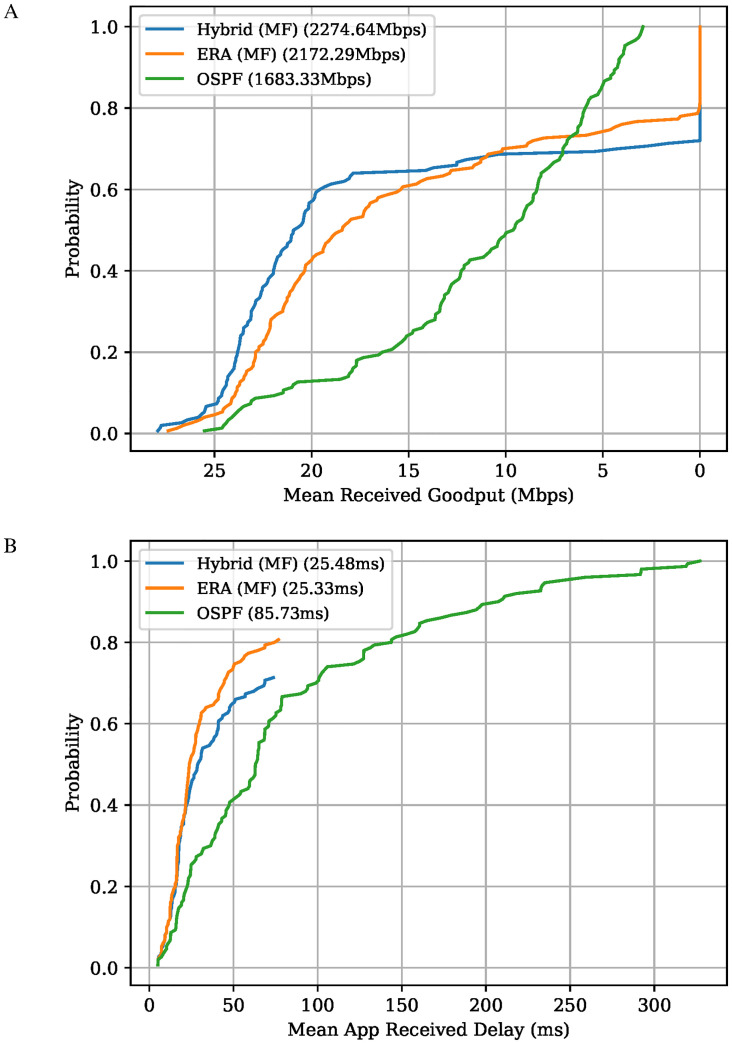
Network performance comparison between the ERA and Hybrid algorithm. MF refers to the solution with the highest network flow value. A: Probability that a flow achieves at least a given average Received Goodput in Mbps, in simulation. The total received Goodput achieved by each algorithm is shown in parentheses. B: Probability that a flow experiences at most a given average end-to-end delay at the application layer, in simulation. The mean application delay achieved by each algorithm is shown in parentheses.

Fig 10 presents the network simulation results for the solutions marked in Fig 9. As expected, the Hybrid Hybrid Maximum Flow (MF) solution has the overall better network *Goodput* performance when compared to all the other points chosen here. OSPF has the worst overall performance both in terms of *Goodput* and mean application delay, even though all the flows are assigned some data rate. Note that OSPF’s delay performance is kept in check thanks to the TCP congestion control mechanism that tries to avoid network congestion. Observe that the ERA MF solution has an overall better delay performance when compared to the Hybrid MF solution. This means that even though the ERA’s *Estimated Mean End-to-End Delay* objective is an approximation, it correlates well with the actual delay performance.

#### Allocated vs actual network performance

**Fig 11:**
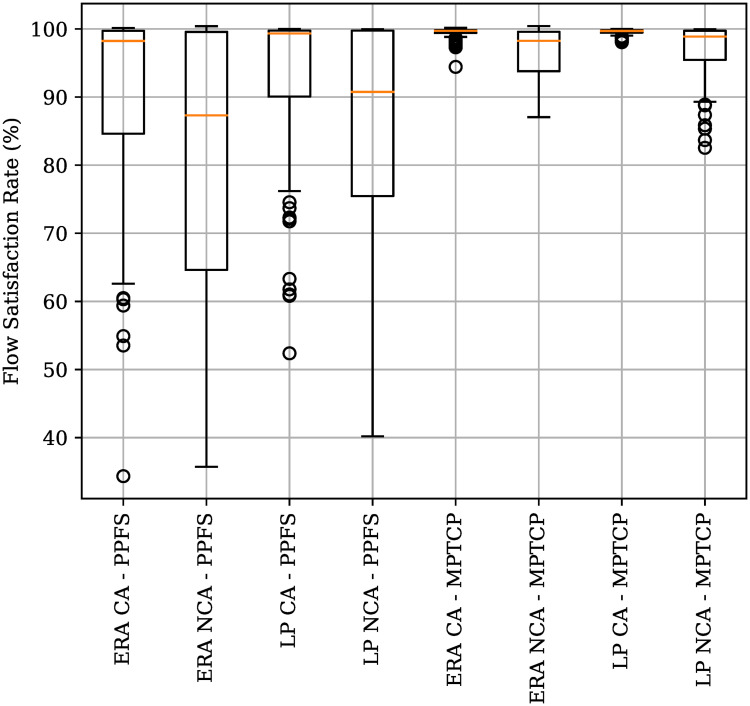
Boxplot showing the distribution of the flow’s satisfaction rate for various multipath routing techniques. MPTCP: MultiPath TCP, PPFS: Per-Packet Flow Split [26], ERA: Evolutionary Routing Algorithm, LP: Linear Programming, CA: Considering Acknowledgements, NCA: Not Considering Acknowledgements.

The main hypothesis surrounding this work is that using routing solutions generated by taking into account the network status leads to an increase in network performance; both in terms of the total throughput carried over the network and resource usage. Given that the routing solution has the network status during its computation, it is to be expected that the network will be devoid of congestion because data rate allocation takes into account the link capacities and all other flows using the network. Therefore, one comes to expect that the rate allocated by the routing algorithm, and the actual data rate seen by the application to be very close to each other. To measure the relationship between the allocated and the actual, the flow satisfaction metric is used. A flow’s satisfaction rate represents the fraction of Goodput received when compared to that allocated by the routing algorithm. For example, a flow allocated 10 Mbps and achieving a throughput of 10 Mbps has a 100% flow satisfaction rate, whereas if it only achieves a throughput of 5 Mbps, the flow satisfaction rate drops to 50%. Fig 11 shows the distribution of the flow’s satisfaction rate for all the flows in a given flow set. The tighter the distribution and the closer it is to 100%, the closer the actual network performance is to the one allocated by the routing solution.

It is well known that TCP and *Per-Packet* multipath routing solutions do not work well with each other. Therefore, it comes as no surprise that when using standard TCP and PPFS, the network performance is nowhere near that promised by the routing algorithm. This can be verified by looking at the columns in Fig 11 that use the PPFS method to deploy *Per-Packet* multipath. What may not be so obvious is the impact the inclusion of acknowledgement flows have on the flow satisfaction rate. Comparing the flow satisfaction rate distribution between the setup when considering acknowledgement rates and when not, the negative effects of ignoring acknowledgements on the flow’s satisfaction rates is clear. Ignoring acknowledgement flows leaves the network open to congestion as soon as acknowledgement flows start. If acknowledgement flows are not taken into consideration when generating the routing solution, not enough capacity may be left in the network links to route this additional traffic. Therefore, as soon as the acknowledgement flows start, some links may be over subscribed. Fig 11 also shows that the modifications done to the MPTCP protocol allow TCP consumer applications to benefit from the advantages offered by a *Per-Packet* multipath routing algorithm with the vast majority of flows having 100% flow satisfaction rate. Even though only one particular flow set is shown in Fig 11, similar conclusions and distributions were seen under different flow sets. Based on these results, we have no reason to believe that the results and conclusions drawn in this section fail to apply under different scenarios than the ones considered here.

#### Effect of path selection algorithm

The path selection affects the properties of the routing solution and the performance that can be extracted from the network. As explained earlier, the KSP algorithm is suited for applications that prioritize delay over throughput, while the opposite is true for the KSREDP algorithm. The KSREDP algorithm is preferred by throughput oriented flows as it has been designed to increase link diversity. As the number of different links available to a flow increases, so does the probability of assigning a flow a higher data rate due to the fact that the paths do not have common links. Having said this, using the KSREDP algorithm is not guaranteed to return better performance than the KSP as this is highly dependent on the network topology and flow set. On the other hand, when using the KSP algorithm, it is guaranteed that the shortest *k* paths are made available to a flow. This means that paths chosen by the KSP algorithm will always outperform or at least match the paths returned by the KSREDP algorithm, in terms of delay. The paths returned by the KSREDP algorithm are not guaranteed to result in solutions that offer higher total network throughput.

**Fig 12:**
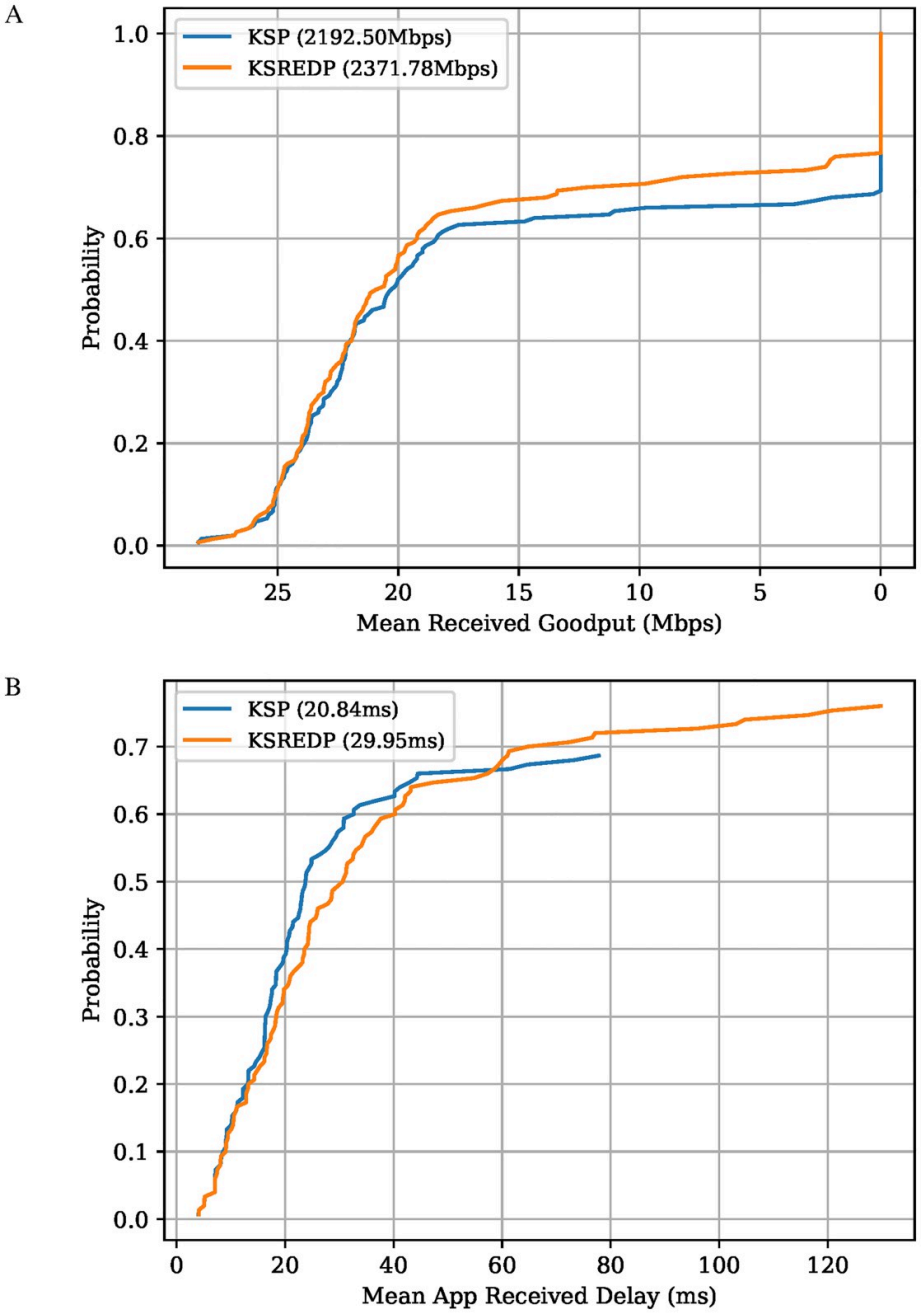
Network performance comparison between the path selection algorithms. A: Probability that a flow achieves at least a given average Received Goodput in Mbps, using network simulations. The total received Goodput achieved by each algorithm is shown in parentheses. The probabilities do not add up to one as unassigned flows are set with a delay value equal to infinity. B: Probability that a flow experiences at most a given average end-to-end delay at the application layer, using network simulations. The mean application delay achieved by each algorithm is shown in parentheses.

The network topology used in this work is highly interconnected; therefore, there is a good chance that the paths found by the KSREDP path selection mechanism do not share many links between them. This link variation brought forward by the KSREDP algorithm allows the routing algorithm to find solutions with a higher amount of total allocated data rate when compared to their KSP counterpart. However, the higher network capacity offered by the KSREDP algorithm comes at the cost of worse delay performance when compared to the KSP algorithm. The network simulation results shown in Fig 12, confirm such statements. The network performance results shown in Fig 12 only use routing solutions to the PC-MFMC problem using LP. Only LP solutions are used here due to LP’s optimality guarantee, such that any performance difference is solely attributed to the different path selection methods used and not the randomness of the algorithm. Based on the results shown in Fig 12, flows that prefer throughput to delay are better off using the KSREDP path selection algorithm. On the other hand, flows that require the best delay performance should favour the KSP algorithm. The scale of the performance difference between the two path selection algorithms relies heavily on the network topology. Developing and testing a solution where each flow gets to choose its own path selection mechanism based on its priorities is an avenue of research worth investigating further.

## Conclusion

The aim of this research has been to increase the efficiency of already deployed networks by increasing throughput and minimizing latency by developing a globally optimized, multipath capable routing algorithm for networks using a centralized network architecture. As a routing algorithm is usually tasked with optimizing for multiple, often conflicting objectives, using LP to find such a solution is not possible. To overcome this limitation, a multi-objective ERA is proposed that makes exclusive use of Per-Packet multipath. Due to the well known negative side effects Per-Packet multipath exhibits when deployed on a network using TCP, TCP is replaced by a modified version of MPTCP. The combination of the modified MPTCP protocol, and the inclusion of the TCP acknowledgement flows when generating a routing solution, guarantee, with a very high probability, that a flow reaches the data rate assigned to it by the routing algorithm. Even though Evolutionary Algorithms (EAs) are inherently suboptimal, the ERA proposed in this work is able to find solutions that are, under all scenarios considered here, on average, 2% off of the optimal solution found using LP.

All the network simulations presented here assume switches are equipped with an infinite buffer size. Network simulations with finite buffers will provide a closer to reality depiction of the performance gap between the devised ERA and the OSPF solution. We conjecture that with the use of finite buffers, the gap between OSPF and the developed system will increase due to the packet drops caused by buffer overflow in times of network congestion. The reason being that TCP treats a packet drop as a sign of congestion and immediately reduces the transmission rate. In the current setup, due to infinite buffers, TCP is allowed to adjust to the ever-increasing RTT.

A limitation of this work is the assumption of a static flow set. For this routing algorithm to be deployed in practice, the routing algorithm has to be updated to handle a dynamic flow set. When using a dynamic flow set, the problem of network instability is one of the main problems that needs to be tackled. To retain network stability, an additional objective needs to be added that minimizes the number of route changes between one routing update to the next. Additionally, the ERA may be set to have the initial population to be equivalent to the final population during the last optimization run. Alternatively, one can use an entirely random population and insert the chosen solution as part of the initial population.

Although no design decision has been made on the basis of the network topology being used here, the fact remains that the systems developed here have been thoroughly tested on a single network topology. Every effort has been made to ensure that the routing algorithm’s design is independent of the topology it is deployed on; however, tests on other network topologies are required to confirm this statement. Having said this, the chosen topology used in this work has enough complexity in terms of number of nodes, links and paths between source-destination pairs that gives us enough confidence to state that with very high probability the solutions and algorithms proposed here will work on other topologies without any major modifications.
